# RAD51 Inhibition Shows Antitumor Activity in Hepatocellular Carcinoma

**DOI:** 10.3390/ijms24097905

**Published:** 2023-04-26

**Authors:** Mingang Pan, Yu Sha, Jianguo Qiu, Yunmeng Chen, Lele Liu, Muyu Luo, Ailong Huang, Jie Xia

**Affiliations:** 1 Key Laboratory of Molecular Biology for Infectious Diseases (Ministry of Education), Chongqing Medical University, Chongqing 400016, China; 2 The First Clinical Medical Institute, Henan University of Chinese Medicine, Zhengzhou 450000, China; 3 Department of Hepatobiliary Surgery, The First Affiliated Hospital, Chongqing Medical University, Chongqing 400016, China

**Keywords:** RAD51, HCC, DNA damage, sorafenib, homologous recombination

## Abstract

Hepatocellular carcinoma (HCC), the major type of liver cancer, causes a high annual mortality worldwide. RAD51 is the critical recombinase responsible for homologous recombination (HR) repair in DNA damage. In this study, we identified that RAD51 was upregulated in HCC and that RAD51 silencing or inhibition reduced the proliferation, migration, and invasion of HCC cells and enhanced cell apoptosis and DNA damage. HCC cells with the combinatorial treatments of RAD51 siRNA or inhibitor and sorafenib demonstrated a synergistic effect in inhibiting HCC cell proliferation, migration, and invasion, as well as inducing cell apoptosis and DNA damage. Single RAD51 silencing or sorafenib reduced RAD51 protein expression and weakened HR efficiency, and their combination almost eliminated RAD51 protein expression and inhibited HR efficiency further. An in vivo tumor model confirmed the RAD51 inhibitor’s antitumor activity and synergistic antitumor activity with sorafenib in HCC. RNA-Seq and gene set enrichment analysis (GSEA) in RAD51-inactivated Huh7 cells indicated that RAD51 knockdown upregulated cell apoptosis and G1/S DNA damage checkpoint pathways while downregulating mitotic spindle and homologous recombination pathways. Our findings suggest that RAD51 inhibition exhibits antitumor activities in HCC and synergizes with sorafenib. Targeting RAD51 may provide a novel therapeutic approach in HCC.

## 1. Introduction

Primary liver cancer causes the third highest cancer-related mortality worldwide and the second highest mortality rate among males. Annually, approximately 906,000 new cases are diagnosed; 830,000 deaths are due to liver cancer, and 75–85% are hepatocellular carcinoma (HCC) [1]. Sorafenib was the only standard chemotherapeutic drug for advanced HCC patients until Lenvatinib was approved in 2018 [2]. Although HCC treatment has improved, the therapeutic effect is unsatisfying. Clinical trials demonstrate that the patients’ median overall survival (OS) under sorafenib increases by only 2.8 months compared with placebo [3]. Therefore, it is important to reveal mechanistic insight into HCC and identify effective drug targets.

DNA damage response (DDR) is a key regulator in maintaining genomic integrity. Genome instability and mutation are new hallmarks of cancer; they activate proto-oncogenes or inactivate tumor suppressor genes, contributing to cancer development [4]. Defects in DDR have been observed in many cancers and contribute to cancer progression [5,6,7]. Regulating DDR pathway genes directly or indirectly provides therapeutic opportunities [8]. The mainstay of cancer chemotherapy has been genotoxic drugs that produce bulky DNA damage which cannot be repaired completely by the DNA damage repair system and results in subsequent cell death [8,9]. PARP inhibitors have been successfully applied to breast cancer, indicating that targeting DNA damage response pathways by exploiting synthetic lethality is a promising therapeutic strategy in cancer [10,11,12]. However, the types of these DDR drugs are limited, and few types apply to HCC.

In this study, we identified a new potential DDR pathway therapeutic target for HCC, RAD51, which is the critical recombinase responsible for homologous recombination (HR) repair in DNA damage. We revealed that RAD51 was upregulated in HCC and that RAD51 silencing or inhibition reduced the proliferation, migration, and invasion of HCC cells and enhanced cell apoptosis and DNA damage. Furthermore, single sorafenib treatment decreased RAD51 protein levels, and inhibition of RAD51 combined with sorafenib exhibited a synergistic antitumor effect. A reduction in RAD51 or single sorafenib treatment resulted in excessive DNA damage and weakened HR efficiency, and their combination almost completely eliminated RAD51 protein expression, further decreased HR efficiency, and increased DNA damage. According to our findings, targeting RAD51 may be a novel therapeutic approach in HCC.

## 2. Results

### 2.1. RAD51 Identified as a Potential DDR Target for HCC, and RAD51 Expression Was Upregulated in HCC

**Figure 1:**
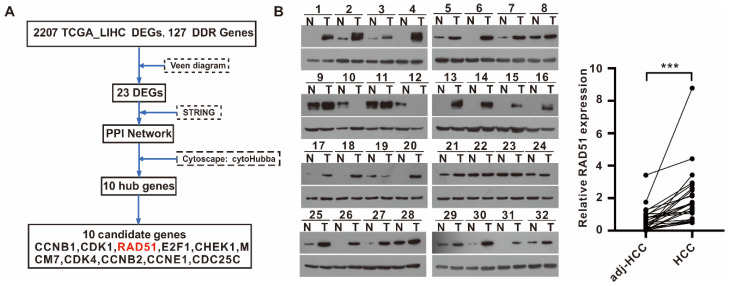
RAD51 identified as a potential DDR target for HCC, and RAD51 expression was upregulated in HCC. (**A**) Identifying 10 candidate drug target genes, including RAD51 in HCC, based on bioinformatics analysis; (**B**) 32 pairs of clinical samples were analyzed for protein levels of RAD51, and the scatter plot shows the relative quantification of RAD51 protein levels. *** *p* < 0.001.

A total of 2207 liver hepatocellular carcinoma (LIHC) differential genes were obtained from the GEPIA database (http://gepia.cancer-pku.cn/, accessed on 10 September 2021) and 127 DDR genes from the PathCards database (https://pathcards.genecards.org, accessed on 10 September 2021) to identify DDR targets for HCC. The intersection of LIHC differential genes and the DDR genes was used to generate a protein–protein interaction (PPI) network (Figure S1A) using STRING (https://string-db.org, accessed on 10 September 2021), and the top 10 hub genes of the PPI network were calculated using the cytoHubba plugin of Cytoscape software (Figure S1B). Then, the 10 hub genes were proposed to be candidate DDR target genes for HCC (Figure 1A). This study focused on RAD51, a key recombinase involved in DNA damage repair, for follow-up experiments. Recently, a bioinformatical study showed RAD51 expression is upregulated in HCC based on the TCGA database and that high RAD51 expression correlates with a poor prognosis [13]. Moreover, we detected RAD51 protein expression in 32 pairs of HCC and adjacent normal tissues using Western blot (WB) assays. The results demonstrated that HCC tissues had a higher RAD51 expression than adjacent normal tissues (Figure 1B). Following that, an immunohistochemistry analysis of five random pairs of HCC tissues confirmed the upregulation of the RAD51 protein in HCC (Figure S1C).

### 2.2. RAD51 Silencing Weakened Cell Proliferation, Migration, and Invasion

**Figure 2:**
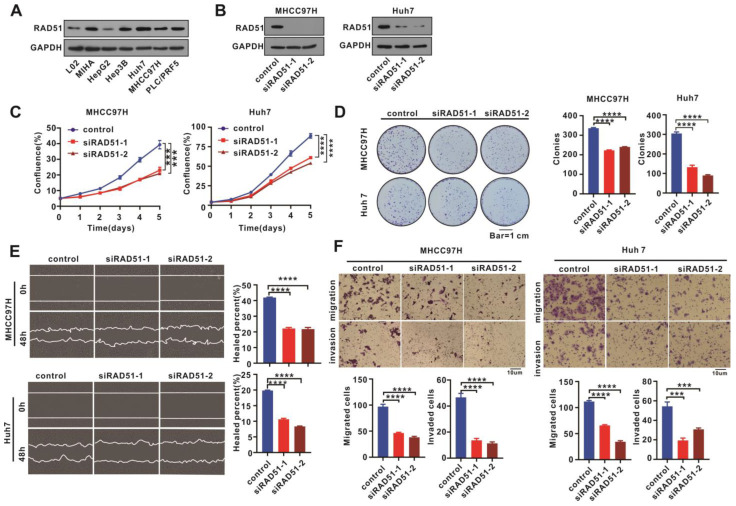
RAD51 silencing weakened cell proliferation, migration, and invasion. (**A**) RAD51 expression was determined in normal liver and HCC cell lines using WB assays. (**B**) MHCC97H and Huh7 cells were treated with siRNA to silence RAD51 expression. (**C**) IncuCyte cell proliferation assays were performed on MHCC97H and Huh7 cells treated with RAD51 siRNA. (**D**) RAD51 silenced or unsilenced cells were grown for two weeks, and the counts of colonies were used to evaluate colony formation capacity. (**E**) Investigating the effect of RAD51 silencing on cell migration using wound healing assays. (**F**) Transwell assays were performed to determine the impact of RAD51 silencing on cell migration and invasion. RAD51 siRNAs at 100 nM was used in these in vitro assays. For (**A**,**B**), the experiment was performed thrice independently, and for (**C**–**F**), each experiment was performed thrice independently with three replicates for each experiment. *** *p* < 0.001, **** *p* < 0.0001.

We detected RAD51 protein expression in normal liver and HCC cell lines using WB assays to understand the role of RAD51 in HCC. We discovered that HCC cell lines expressed RAD51 protein at higher levels than the normal liver cell line (L02, MIHA) but not HepG2 (Figure 2A). Then, high-RAD51-expressing MHCC97H and Huh7 cells were selected to silence RAD51 expression using RAD51 siRNA (Figure 2B), and the effect of the RAD51 silencing was investigated in vitro using different assays. IncuCyte cell proliferation and colony formation assays were performed to evaluate the RAD51 silencing on cell proliferation and colony formation ability, respectively. According to the results, RAD51 silencing reduced MHCC97H and Huh7 cell proliferation and their capacity to form colonies (Figure 2C,D). Furthermore, we tested cell migration and invasion abilities using wound healing and transwell assays. Based on the result of the wound healing assay, RAD51 silencing weakened cell migration ability (Figure 2E), while the transwell assay further confirmed that RAD51 silencing inhibited the MHCC97H and Huh7 cells’ migration and invasion capabilities (Figure 2F).

### 2.3. RAD51 Silencing Enhanced Cell Apoptosis and DNA Damage

**Figure 3:**
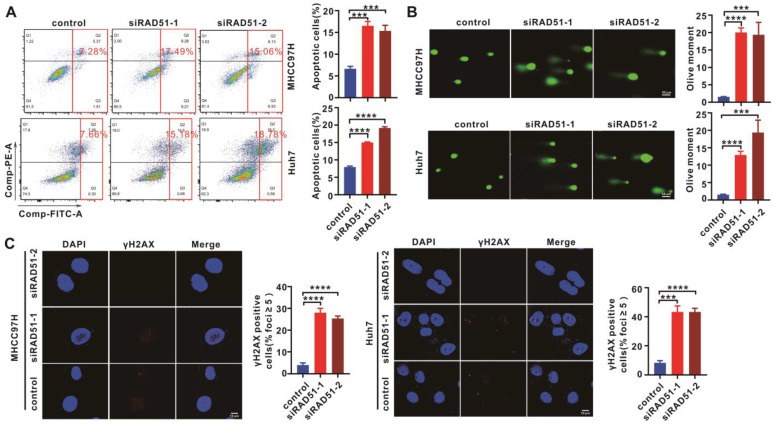
RAD51 silencing increased cell apoptosis and DNA damage. (**A**) Investigating the effect of RAD51 silencing on cell apoptosis of MHCC97H and Huh7 cells. (**B**) Comet assays were performed to determine the effect of RAD51 silencing on DNA damage. (**C**) The effect of RAD51 silencing on DNA damage was evaluated using immunofluorescence of γH2AX. RAD51 siRNAs at 100 nM were used in these in vitro assays. Each experiment was performed thrice independently with three replicates for each experiment. *** *p* < 0.001, **** *p* < 0.0001.

Cell apoptosis was detected after RAD51 silencing, and the results demonstrated that RAD51 silencing induced cell apoptosis in MHCC97H and Huh7 cells (Figure 3A). Considering that RAD51 plays a central role in homologous-recombination-mediated DNA double-strand break repair, the DNA damage level was detected using a comet assay. The results indicated that DNA damage increased in RAD51-silenced MHCC97H and Huh7 cells (Figure 3B). Then, γH2AX, a DNA double-strand break marker, increased after RAD51 silencing, measured using an immunofluorescence assay (Figure 3C).

### 2.4. RAD51 Silencing Displays a Synergistic Antitumor Effect with Sorafenib

**Figure 4:**
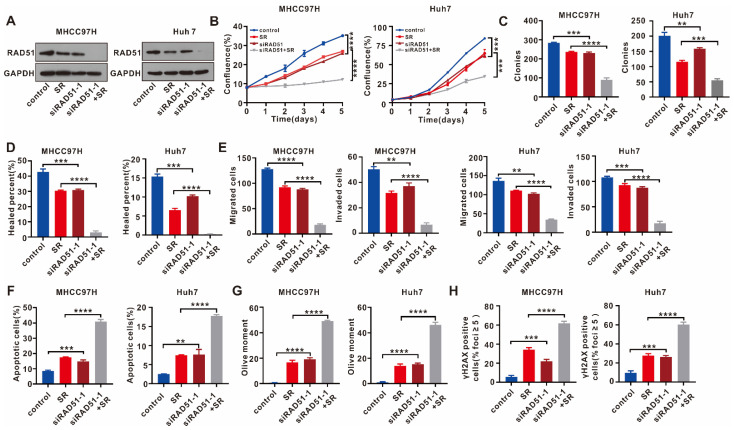
RAD51 silencing shows a synergistic antitumor effect with sorafenib. (**A**) The combined effect of RAD51 silencing with sorafenib on RAD51 protein levels was detected using WB assays. (**B**) The effect of RAD51 silencing combined with sorafenib on cell proliferation of HCC cells was determined using the IncuCyte cell proliferation assay. (**C**) Investigating the combined effect of RAD51 silencing and sorafenib on HCC cells’ colony formation ability using colony formation assays. (**D**) Wound healing assays were used to evaluate the effect of RAD51 silencing combined with sorafenib on cell migration. (**E**) The effect of RAD51 silencing combined with sorafenib on cell migration and invasion was detected using transwell assays. (**F**) Cell apoptosis was analyzed to evaluate the effect of RAD51 silencing combined with sorafenib on HCC cells. (**G**) DNA damage of HCC cells treated with sorafenib or RAD51 siRNA was examined using the comet assay. (**H**) The level of γH2AX was examined in MHCC97H and Huh7 cells treated with sorafenib or RAD51 siRNA using immunofluorescence. In these in vitro assays, MHCC97H cells were treated with sorafenib at 5 μM and/or RAD51 siRNA-1 at 50 nM, and Huh7 cell were treated with sorafenib at 5 μM and/or RAD51 siRNA-1 at 50 nM. For (**A**,**B**), the experiment was performed thrice independently, and for (**C**–**H**), each experiment was performed thrice independently with three replicates for each experiment. ** *p* < 0.01, *** *p* < 0.001, **** *p* < 0.0001.

Sorafenib is the standard treatment for advanced HCC patients. Surprisingly, single sorafenib (SR) treatment decreased RAD51 expression at protein levels (Figure 4A) but not mRNA expression (Figure S2A), and the combination of sorafenib and RAD51 silencing almost completely eliminated RAD51 protein expression (Figure 4A). We then examined the effect of sorafenib combined with RAD51 silencing on HCC cells in vitro to determine whether RAD51 silencing synergizes with sorafenib. Based on the results, sorafenib and RAD51 silencing had a synergistic antitumor effect on MHCC97H and Huh7 cells. According to IncuCyte cell proliferation and colony formation assays, the combination treatment of sorafenib and RAD51 silencing further inhibited cell proliferation and colony formation capacity (Figure 4B,C). Wound healing and cell migration and invasion assays in transwells showed that the combination treatment of sorafenib and RAD51 silencing attenuated cell migration and invasion further (Figure 4D,E). In the cell apoptosis analysis, the combination treatment further increased cell apoptosis (Figure 4F). The comet assay and immunofluorescence of γH2AX confirmed that DNA damage was further induced under the combination treatment (Figure 4G,H).

### 2.5. IBR2 Reduced Cell Proliferation, Migration, and Invasion and Increased Apoptosis and DNA Damage

**Figure 5:**
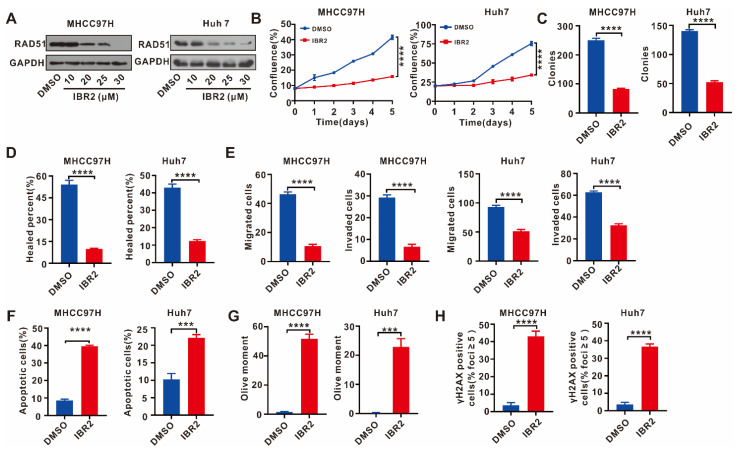
IBR2 decreased cell proliferation, migration, and invasion and increased apoptosis and DNA damage. (**A**) The RAD51 protein expression in MHCC97H and Huh7 cells treated with various concentrations of IBR2 were examined using WB assay. (**B**) IncuCyte cell proliferation assays were performed on cells treated with IBR2. (**C**) Cells treated with IBR2 were grown for two weeks, and the colonies were captured and counted. (**D**) Wound healing assays were used to investigate the effect of IBR2 on cell migration. (**E**) Transwell assays were conducted to assess the effect of IBR2 on cell migration and invasion. (**F**) Cell apoptosis of HCC cells was analyzed using flow cytometry after treatment of IBR2. (**G**) DNA damage was examined on MHCC97H and Huh7 cells treated with IBR2 using the comet assay. (**H**) The γH2AX levels were examined in HCC cells treated with IBR2 using immunofluorescence. MHCC97H and Huh7 cells were treated with IBR2 at 30 μM in these assays. For (**A**), the experiment was performed thrice independently, and for (**B**–**H**), each experiment was performed thrice independently with three replicates for each experiment. *** *p* < 0.001, **** *p* < 0.0001.

We treated MHCC97H and Huh7 cells with the RAD51 inhibitor IBR2, which can induce the ubiquitination and degradation of RAD51 [14]. IBR2 decreased RAD51 protein expression in MHCC97H and Huh7 cells after being treated with various concentrations of IBR2 (Figure 5A). Then, we observed how IBR2 affects cell function, and the results were identical to those observed in RAD51-silenced cells. The IncuCyte cell proliferation and colony formation assays demonstrated that IBR2 weakened the cell proliferation of MHCC97H and Huh7 cells and reduced the colonies (Figure 5B,C). Then, a wound healing assay showed that IBR2 attenuated the cell migratory capability of MHCC97H and Huh7 cells (Figure 5D). Transwell assays confirmed that IBR2 attenuated the cell migratory and invasive ability of MHCC97H and Huh7 cells (Figure 5E). Cell apoptosis detection showed IBR2 increased cell apoptosis levels (Figure 5F). The comet and γH2AX immunofluorescence assays demonstrated that IBR2 increased DNA damage and γH2AX foci (Figure 5G,H). Then, we treated MHCC97H and Huh7 cells with IBR2 and sorafenib. The results suggested that the combination treatment of sorafenib and IBR2 further reduced cell proliferation, migration, and invasion while increasing cell apoptosis and DNA damage (Figure S3).

### 2.6. Targeting RAD51 Inhibited HR Activity In Vitro, Attenuated Tumor Growth In Vivo, and Exhibited a Synergistic Antitumor Effect with Sorafenib

**Figure 6:**
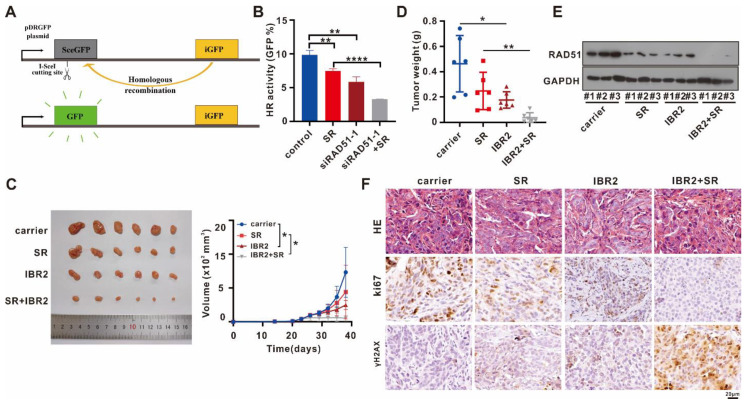
IBR2 suppressed tumor growth in vivo and exhibited synergistic antitumor activity with sorafenib. (**A**) Schematic representation of HR assay. The pDRGFP plasmid contains two different GFPs: a mutated GFP (grey) containing an I-SceI endonuclease site and iGFP (yellow); the I-SceI site can be repaired by HR using the iGFP template, resulting in a functional GFP (green). (**B**) 293T cells transfected with sicontrol or siRAD51, together with pDRGFP+I-SceI, treated with or without sorafenib, were analyzed by flow cytometry to test GFP percentage (GFP%). 293T cells were treated with sorafenib at 5 μM and/or RAD51siRNA at 50 nM. Experiment was performed thrice independently with three replicates. (**C**) IBR2 and sorafenib inhibited MHCC97H xenograft tumors, and the combined therapy of IBR2 and sorafenib inhibited tumor growth. (**D**) The weight of xenograft tumors was measured when mice were sacrificed. (**E**) RAD51 protein levels in xenograft tumors were examined using WB assays. (**F**) Xenograft tumors stained with HE, Ki67, and H2AX are shown. Experiment was performed thrice independently with three replicates. * *p* < 0.05, ** *p* < 0.01, **** *p* < 0.0001.

RAD51 is the key recombinase of the HR repair system. We observed HR activity in 293T cells transfected with RAD51 siRNA or treated with sorafenib using a pDR-GFP system (Figure 6A). We discovered that single RAD51 silencing or sorafenib inhibited HR activity, and the combination of RAD51 silencing and sorafenib suppressed HR activity further (Figure 6B). We implanted MHCC97H cells into nude mice and observed them every three days to test the antitumor activity by targeting RAD51 in vivo. After the average tumor size reached 5 mm (length) × 5 mm (width), all mice were injected with IBR2 or sorafenib and their combined therapy for 2 weeks (twice a week, 10 mg/kg IBR2 and 10 mg/kg sorafenib). After two weeks of therapy, all nude mice were sacrificed. In mice treated with IBR2 or sorafenib, the tumors grew slower (Figure 6C), and the average tumor weight was lower than in the control mice (Figure 6D). Sorafenib and IBR2 combined therapy lowered the tumor volume and weight compared with single IBR2 or sorafenib therapy (Figure 6C,D). WB assay indicated that single sorafenib or IBR2 decreased RAD51 protein levels, and the combination of sorafenib and IBR2 almost completely eliminated RAD51 protein expression (Figure 6E). Figure 6F presents hematoxylin and eosin (HE), ki67, and γH2AX staining of nude xenograft tumors. The results confirmed the antitumor activities by targeting RAD51 in HCC.

### 2.7. Gene Set Enrichment Analysis (GSEA) Confirmed the Antitumor Activity of Inactivated RAD51

**Figure 7:**
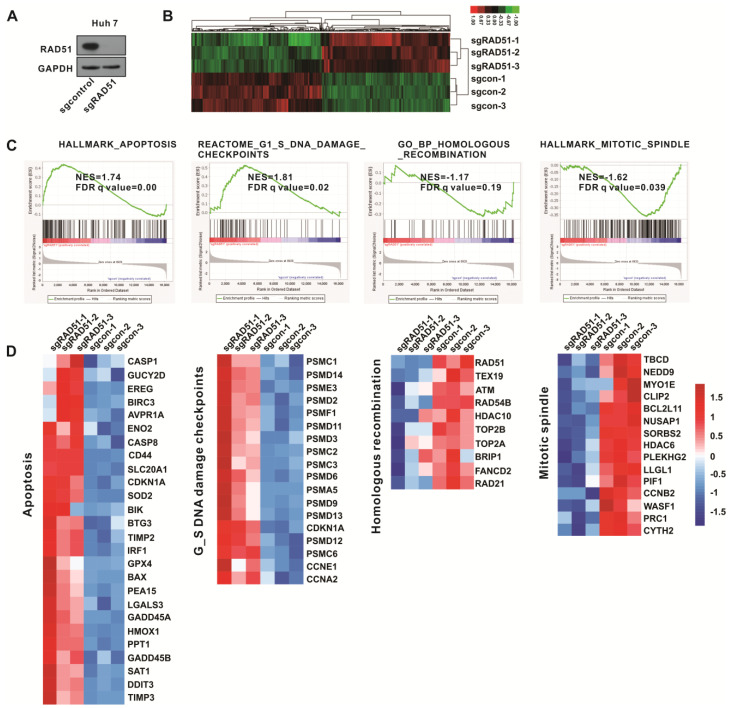
Gene set enrichment analysis confirmed the antitumor activity of inactivated RAD51. (**A**) The RAD51 protein expression in RAD51-inactivated Huh7 cells using CRISPR/Cas9 technology. (**B**) Heatmap of all DEGs of Huh7 cells after RAD51 inactivation. (**C**) GSEA analysis predicated that inactivated RAD51 upregulates apoptosis and G1/S DNA damage checkpoint pathways and downregulates homologous recombination and mitotic spindle pathways. (**D**) Heatmaps of the genes enriched in indicated pathways demonstrate gene expression changes upon RAD51 knockdown. Signals in red represent higher expression levels, while blue signals indicate lower levels based on the group’s mean expression level.

To further confirm the antitumor activity of targeting RAD51, RAD51 was inactivated in Huh7 cells using CRISPR/Cas9 technology (Figure 7A). The RNA sequencing assay demonstrated that there are 734 differentially expressed genes between Huh7 sgcontrol and Huh7 sgRAD51 with a fold change of ≥1.5 and *p* < 0.05 (Figure 7B). GSEA suggested that RAD51 inactivation upregulated cell apoptosis and G1/S DNA damage checkpoint pathways while downregulating homologous recombination and mitotic spindle pathways (Figure 7C). These results confirmed the antitumor activity of targeting RAD51. The top-scoring genes altered in the four gene sets included many genes associated with cell apoptosis and DNA damage, such as GADD45A, DDIT3, BAX, CASP1, CASP8, ATM, and FANCD2 (Figure 7D), demonstrating that diminishment of RAD51 contributes to the tumor suppressive effect.

## 3. Discussion

HCC is a commonly emerging and diagnosed cancer worldwide with a high mortality rate due to the lack of effective therapies. Surgical and locoregional therapies are the primary treatment approaches for the early/intermediate HCC stage, while systemic therapy is the only option for advanced unresectable HCC [3,15,16]. Identifying novel pharmacological targets of HCC and developing new systemic therapeutic drugs is important. Our study indicates that RAD51 is frequently upregulated in HCC. RAD51 silencing or pharmacological inhibition demonstrated antitumor activity in cancer cell progression and enhanced cell apoptosis and DNA damage. Furthermore, sorafenib treatment decreased RAD51 protein expression and HR activity, and RAD51 silencing or inhibition had a synergistic inhibitory effect with sorafenib in HCC. Therefore, our study indicated that targeting RAD51 might be a novel and potent therapeutic approach for HCC.

RAD51 has an N-terminal domain containing five α-helices, a β-strand, two disordered loops that bind DNA, and a flexible interdomain linker [17]. Previous studies demonstrated that RAD51 performs homology search and strand invasion during homologous recombination repair for DNA damage [18,19,20]. RAD51 has been frequently found to be upregulated in many cancers and correlated with poor survival and chemotherapy resistance [21,22,23,24,25]. RAD51 overexpression may result in DNA hyperrecombination, contributing to genomic instability, and might transform normal cells into cancer cells, influencing cancer progression [26,27]. Targeting RAD51 inhibition has demonstrated an antitumor effect in several cancers [28,29,30,31]. Conversely, previous studies suggested that RAD51 overexpression contributed to genome instability [26] and induced slow-growth phenotypes and increased apoptosis in HT1080 human fibrosarcoma cells [32]. However, the role of RAD51 in HCC remains unknown. Recently, Xu et al. reported that RAD51 expression correlated with immune infiltration and that targeting RAD51 might be a potential immunotherapy target in HCC [13]. In our study, the data suggested that RAD51 was upregulated in HCC, RAD51 silencing or inhibition exhibits antitumor activity toward HCC, and RAD51 inhibition has a synergistic inhibitory effect with sorafenib. 

Sorafenib is a multitarget inhibitor for suppressing tumor angiogenesis and proliferation, the targets including some serine/threonine kinases, such as C-Raf, wildtype B-Raf, and mutant B-Raf, as well as some receptor tyrosine kinases, such as VEGFR 1-3, PDGFR, and c-KIT [33]. DNA damage accumulated in tumor cells beyond the capacity of DNA damage repair system leads to cell apoptosis [34,35], and most drugs used clinically are designed based on this principle [9,36]. Wang et al. have reported that the induction of DNA damage by CDK12 inhibition is synergistic with sorafenib treatment in HCC [37], and it shows that the induction of DNA damage combined with sorafenib treatment may be a more effective therapeutical approach in HCC. However, the role of sorafenib in the induction of DNA damage remains unknown. In our study, sorafenib treatment reduced RAD51 protein expression and increased subsequent DNA damage and cell apoptosis. When RAD51 downregulation was combined with sorafenib, the DNA damage and cell apoptosis increased further. This may explain the antitumor activity by targeting RAD51 and the synergistic antitumor activity by combining RAD51 inhibition with sorafenib. Single sorafenib treatment decreased RAD51 protein levels and HR activity in our results, suggesting that RAD51 and HR may be the downstream targets of sorafenib. This study is the first to report that sorafenib can suppress RAD51 expression and HR activity. The details of how sorafenib affects homologous recombination and RAD51 protein expression need further investigation. Recently, Samadaei et al. reported that B02, another RAD51 inhibitor, in combination with sorafenib significantly enhanced the efficacy of sorafenib in reducing the cell viability, colony formation ability, and invasion capacity of HCC cells [38]. These findings are in agreement with our results. Moreover, we overexpressed RAD51 in HepG2 cells with low RAD51 levels, and the RAD51 overexpression promoted cell proliferation, migration, or invasion (Figure S4), identical to a previous study in esophageal squamous cell carcinoma [39].

In summary, we discovered that RAD51 was upregulated in HCC, and RAD51 silencing or inhibition exhibited antitumor and synergistic antitumor activities with sorafenib toward HCC by increasing bulky intracellular DNA damage. Our study suggests that RAD51 may be a therapeutic target for HCC. Future studies are warranted to investigate the function of RAD51 in HCC.

## 4. Materials and Methods

### 4.1. Bioinformatics Analysis

The Cancer Genome Atlas liver hepatocellular carcinoma differential genes (TCGA_LIHC DEGs) were obtained from the Gene Expression Profiling Interactive Analysis (GEPIA) database (http://gepia.cancer-pku.cn/, accessed on 10 September 2021). DDR genes were acquired from the PathCards database (https://pathcards.genecards.org/, accessed on 10 September 2021). The intersection of TCGA_LIHC DEGs and DDR genes was calculated using an online Venn diagram (http://bioinformatics.psb.ugent.be/webtools/Venn/, accessed on 10 September 2021). The PPI network was built using the STRING online database (https://string-db.org/, accessed on 10 September 2021). Hub genes were determined using cytoHubba in the Cytoscape software (http://cytoscape.org, accessed on 10 September 2021). Gene set enrichment analysis software (GESA, v4.1.0, available at www.broadinstitute.org/gsea, accessed on 6 June 2022) and gene sets from the molecular signatures database (https://www.gsea-msigdb.org/gsea/msigdb/index.jsp, accessed on 15 June 2022) were used to perform GSEA analysis.

### 4.2. Clinical Patient Specimens

Twenty-eight pairs of HCC and paracancerous tissues were obtained from the First Affiliated Hospital of Chongqing Medical University for Western blotting (WB) assay. For immunohistochemistry (IHC), five pairs of HCC and matching paracancerous tissues were embedded in paraffin for subsequent experiments.

### 4.3. Cell Culture and siRNA Transfection

HCC cell lines PLC/PRF/5 and Huh7 were purchased from ATCC (Rockville, MD, USA). Human normal liver cell lines LO2 and MIHA and HCC cell lines MHCC97H, HepG2, and Hep3B were obtained from the Liver Cancer Institute, Zhongshan Hospital, Fudan University (Shanghai, China). Dulbecco’s modified eagle medium (DMEM, HyClone, Logan, UT, USA) containing 10% fetal bovine serum (FBS) and 1% penicillin/streptomycin (HyClone, Logan, UT, USA) was used as the culture medium. The cells were grown in a humidified 5% CO_2_ incubator at 37 °C. When specified, siRNA (Tsingke, Beijing, China) was transfected into cells at 50–100 nM using LipoRNAi reagent (Beyotime, Shanghai, China). Fresh medium was replaced 6 h after transfection, cells were collected at 24 h or 48 h for RNA extraction and 48 h for WB assays, and other experiments were conducted 24 h later after transfection.

### 4.4. RNA Extraction and Quantitative Reverse Transcription (RT-qPCR)

Total RNA from cells was extracted with TRIzol reagent (Thermo Fisher Scientific, Waltham, MA, USA), and a Takara reverse transcription kit (Takara, Kusatsu, Shiga, Japan) was used to perform reverse transcription. Next, RT-qPCR was conducted using SYBR green mix (Bimake, Houston, TX, USA) in Bio-Rad’s CFX Connect real-time system (Bio-Rad, Hercules, CA, USA). The cycle times (Ct value) of genes were normalized to the β-actin value in the same sample. The relative gene expression was analyzed using the 2-ΔΔCt method.

### 4.5. siRNA

The siRNAs consisted of a negative control (TSINKE, Beijing, China), RAD51#1 siRNA-F-CGAUGUGAAGAAAUUGGAATT siRNA-R-UUCCAAUUUCUUCACAUCGTT, and RAD51#2 siRNA-F-GACUGGAUCUAUCACAGAATT siRNA-R-UUCUGUGAUAGAUCCAGUCTT.

### 4.6. WB Assay

RIPA lysis buffer (CWBIO, Beijing, China) was used to isolate total protein from cells or tissues on ice; for isolation of tissue protein, tissues were homogenized using the suggested homogenization protocols of the FastPrep-24TM 5G homogenizer (MP Biomedicals, Santa Ana, CA, USA), and then all samples were centrifuged at 4 °C, 12,000 rpm for 20 min. BCA protein assay kit (CWBIO, Beijing, China) was used to perform protein concentration quantification. Then, Western blotting (WB) assays of all protein samples were performed according to the Abcam-recommended WB protocol (https://www.abcam.cn/protocols/general-western-blot-protocol-2, accessed on 15 September 2019) using Bio-Rad Gel Electrophoresis Systems (Bio-Rad, Hercules, CA, USA). A quantitative analysis of WB bands was conducted using ImageJ (NIH, Bethesda, ML, USA).

### 4.7. Antibodies and Reagents

All antibodies and reagents are listed in Table 1.

### 4.8. Immunohistochemistry (IHC)

The ABC Peroxidase Staining Kit (Thermo Fisher Scientific, Waltham, MA, USA) was used for IHC staining. Briefly, paraffin-embedded tissues were cut into 4 μm sections, and deparaffinization was conducted in ethanol and xylene. Antigen repair was performed in a pressure cooker, and endogenous peroxidase activity was inactivated with 3% H_2_O_2_. Then, all sections were blocked with goat serum, and the primary antibody was incubated overnight at 4 °C. For protein visualization, sections were stained with ABC Peroxidase Staining Kit (Thermo Fisher Scientific, Waltham, MA, USA) and DAB Detection kit (ZhongShanJinQiao, Beijing, China) after incubation with the secondary antibody.

### 4.9. IncuCyte Cell Proliferation Assay

Cells were seeded in 96-well plates (1000–2000 cells per well) and imaged using IncuCyte ZOOM (Essen Bioscience, Ann Arbor, MI, USA) every 24 h. After 120 h, cell confluence was used to identify cell proliferation using phase-contrast images.

### 4.10. Colony Formation Assay

The cells were seeded in 6-well plates (1000 cells per well). On day 14, paraformaldehyde was used for fixation, and crystal violet (Beyotime, Shanghai, China) was used for staining. Then, the number of cell colonies was counted to evaluate cell proliferation.

### 4.11. Wound Healing Assay

Cells were plated in 12-well plates; then, a 10 μL plastic pipette tip was used for scratching monolayers. The phase-contrast images were captured at 0 and 48 h using IncuCyte ZOOM (Essen Bioscience, Ann Arbor, MI, USA). After 48 h, these images were collected and analyzed using ImageJ software (NIH, Bethesda, ML, USA) to evaluate cell migration ability.

### 4.12. Transwell Cell Migration and Invasion Assays

The 24-well Corning plates (8 μm pore size) were used to assess cell migration and invasion. For migration assays, the upper chambers were filled with 2.5–5 × 10^4^ cells in DMEM without FBS, while DMEM containing 10% FBS was used for the lower chambers. For invasive assays, the upper chambers were preincubated with 100 µL BD Matrigel (200–300 µg/mL diluted with FBS-free DMEM, BD Biosciences, Franklin Lakes, NJ, USA) at 37 °C in a 5% CO_2_ humidified atmosphere for 2 h. Then, the upper chambers were filled with 2.5–5 × 10^4^ cells in DMEM without FBS, while DMEM with 10% FBS was used for the lower chambers. Compounds, such as IBR2 and sorafenib, were added to the upper and lower chambers at the same concentration. After 48 h, the cells on the top side of each upper chamber were removed by scratching using wet cotton swabs. Fixation and staining were conducted with 4% paraformaldehyde and 1% crystal violet staining solution, respectively (Beyotime, Shanghai, China). Then, the number of cells outside the upper chamber was counted to evaluate cell migratory or invasive ability.

### 4.13. Cell Apoptosis Analysis

A PE/FITC apoptosis kit (BD, Biosciences, Franklin Lakes, NJ, USA) was employed to detect cell apoptosis. Briefly, cells were collected and stained with 5 µL PE and 5 µL FITC for 15 min, and then cell apoptosis levels were determined using flow cytometry (BD Biosciences, Franklin Lakes, NJ, USA).

### 4.14. Immunofluorescence Assay 

HCC cells were plated on culture slides in 12-well plates. After incubation for 24 h, cells were fixed with 4% paraformaldehyde for 20 min and permeabilized with 0.1% Triton X-100 for 10 min. Then, cells were incubated with primary antibody overnight at 4 °C and stained with Alexa Fluor 594 goat anti-rabbit IgG for 1 h at 37 °C. The cells were visualized and photographed in a laser scanning confocal microscope (Leica, Wetzlar, Germany) after 4′,6-diamidine-2-phenyl indole (DAPI) staining to indicate the nucleus. γH2AX foci were quantified using the FindFoci plugin in ImageJ [40] (NIH, Bethesda, ML, USA).

### 4.15. Comet Assay

A comet assay kit (Wanleibio, Shenyang, China) was used to detect DNA damage. Briefly, HCC cells were mixed with 2% agarose at a 1:1 ratio, plated on slides precoated with 0.5% agarose, lysed for 1.5 h in lysis buffer, electrophoresed for 25 min at 25 V, and then analyzed and photographed using a fluorescence microscope (Leica, Wetzlar, Germany). Images acquired were analyzed using the Open Comet software, and Olive moment was generated to evaluate DNA damage [41].

### 4.16. Plasmid DNA Construction and Lentivirus Packaging

LentiCRISPR-v2, pMD2.G, and psPAX2 plasmids were provided by Prof. Ni Tang (Key Laboratory of Molecular Biology on Infectious Diseases, Chongqing Medical University). The E-CRISP website (http://www.e-crisp.org/ECRISP/designcrispr.html, accessed on 16 August 2020) was used to obtain the RAD51-targeting sequences (sgRAD51-F-CACCGTGATCTCTGACCGCCTTTGG, sgRAD51-R-AAACCCAAAGGCGGTCAGAGATCAC). The RAD51 sgRNA was embedded in lentiCRISPR-v2. The pLVML-3×FLAG-MCS-Puro plasmid was purchased from Hunan Fenghui Biotechnology (Changsha, China). A PCR-amplified fragment of the RAD51 CDS (coding sequence) was cloned into the MCS domain of the expression plasmid. Then, 3 µg lentiviral vectors were packaged using lipo8000-based (Beyotime, Shanghai, China) cotransfection of HEK293T cells with 2 µg psPAX2 and 1 µg pMD2.G.

### 4.17. HR Assays

The HR assays were conducted according to previous reports [42]. Briefly, the 293T cells were transfected with pDRGFP and I-SceI expression vectors. After 48 h, a Beckman Coulter flow cytometer (Beckman Coulter, Brea, CA, USA) was used to determine the HR-mediated repair activity by analyzing the GFP positive cells (GFP%) in approximately 10,000 cells.

### 4.18. RNA Sequencing

Cells were harvested with TRIzol reagent (Invitrogen, Waltham, MA, USA) for total RNA extraction. cDNA libraries were prepared for each pooled RNA sample using Ion Total RNA-Seq Kit v2.0 (Life Technologies, Carlsbad, CA, USA). For proton sequencing, cDNA libraries were processed using commercial protocols. RNA sequencing experiments were performed by NovelBio Corp. Laboratory (Shanghai, China).

### 4.19. Animal Models

Male BALB/c nude mice were purchased from ENSIWEIER Corporation (ChongQing, China); 6 nude mice per group were subcutaneously injected with 5 × 10^6^ MHCC97H cells in 100 µL PBS, and the mice were observed every 3 days. After the average tumor size reached 5 mm (length) × 5 mm (width), mice were injected with IBR2 and sorafenib for 2 weeks (twice a week, 10 mg/kg IBR2 and 10 mg/kg sorafenib). Mice were sacrificed after treatment, and tumor size and weight were measured.

### 4.20. Statistical Analysis

Data analysis was carried out with GraphPad Prism 8.3.0. Values are presented as mean ± SD.

## Figures and Tables

**Table 1:** Antibodies and reagents.

Catalog	Antibody/Regent	Antibody Species	MW (KD)	Company	Application and Radio
Ab133534	RAD51	R	37	Abcam	WB 1:10,000 IHC 1:200
TA-08	GAPDH	M	37	ZSGB-BIO	WB 1:2000
9718	γH2AX	R	15	CST	IF IHC 1:250
PA5-16785	Ki67	M		Invitrogen	IHC 1:200
**Catalog**	**Regent**			**Company**	
HY-103710 S7397 WLA123a	IBR2			MCE	
	Sorafenib			Selleck	
	Comet assay kit			Wanlei	

## Data Availability

The RNA sequencing analysis data used in this study can be found in the GEO database (GSE216728).

## Supplementary Materials

The following supporting information can be downloaded at: https://www.mdpi.com/article/10.3390/ijms24097905/s1.

## References

[1] Sung H., Ferlay J., Siegel R.L., Laversanne M., Soerjomataram I., Jemal A., Bray F. (2021). Global Cancer Statistics 2020: GLOBOCAN Estimates of Incidence and Mortality Worldwide for 36 Cancers in 185 Countries. CA Cancer J. Clin..

[2] Lenvatinib versus Sorafenib in First-Line Treatment of Patients with Unresectable Hepatocellular Carcinoma: A Randomised Phase 3 Non-Inferiority Trial-PubMed. https://pubmed.ncbi.nlm.nih.gov/29433850/.

[3] Huang A., Yang X.-R., Chung W.-Y., Dennison A.R., Zhou J. (2020). Targeted Therapy for Hepatocellular Carcinoma. Signal. Transduct. Target Ther..

[4] Hanahan D., Weinberg R.A. (2011). Hallmarks of Cancer: The next Generation. Cell.

[5] Loss of Ribonuclease DIS3 Hampers Genome Integrity in Myeloma by Disrupting DNA:RNA Hybrid Metabolism|The EMBO Journal. https://www.embopress.org/doi/full/10.15252/embj.2021108040.

[6] SMG8/SMG9 Heterodimer Loss Modulates SMG1 Kinase to Drive ATR Inhibitor Resistance | Cancer Research | American Association for Cancer Research. https://aacrjournals.org/cancerres/article/82/21/3962/709958/SMG8-SMG9-Heterodimer-Loss-Modulates-SMG1-Kinase.

[7] Jurkovicova D., Neophytou C.M., Gašparović A.Č., Gonçalves A.C. (2022). DNA Damage Response in Cancer Therapy and Resistance: Challenges and Opportunities. Int. J. Mol. Sci..

[8] Therapeutic Opportunities within the DNA Damage Response-PubMed. https://pubmed.ncbi.nlm.nih.gov/25709118/.

[9] O’Connor M.J. (2015). Targeting the DNA Damage Response in Cancer. Mol. Cell.

[10] Anders C.K., Winer E.P., Ford J.M., Dent R., Silver D.P., Sledge G.W., Carey L.A. (2010). Poly(ADP-Ribose) Polymerase Inhibition: “Targeted” Therapy for Triple-Negative Breast Cancer. Clin. Cancer Res..

[11] Papadimitriou M., Mountzios G., Papadimitriou C.A. (2018). The Role of PARP Inhibition in Triple-Negative Breast Cancer: Unraveling the Wide Spectrum of Synthetic Lethality. Cancer Treat. Rev..

[12] Mateo J., Lord C.J., Serra V., Tutt A., Balmaña J., Castroviejo-Bermejo M., Cruz C., Oaknin A., Kaye S.B., de Bono J.S. (2019). A Decade of Clinical Development of PARP Inhibitors in Perspective. Ann. Oncol..

[13] Xu H., Xiong C., Chen Y., Zhang C., Bai D. (2021). Identification of Rad51 as a Prognostic Biomarker Correlated with Immune Infiltration in Hepatocellular Carcinoma. Bioengineered.

[14] Zhu J., Zhou L., Wu G., Konig H., Lin X., Li G., Qiu X., Chen C., Hu C., Goldblatt E. (2013). A Novel Small Molecule RAD51 Inactivator Overcomes Imatinib-resistance in Chronic Myeloid Leukaemia. EMBO Mol. Med..

[15] Llovet J.M., Montal R., Sia D., Finn R.S. (2018). Molecular Therapies and Precision Medicine for Hepatocellular Carcinoma. Nat. Rev. Clin. Oncol..

[16] Gordan J.D., Kennedy E.B., Abou-Alfa G.K., Beg M.S., Brower S.T., Gade T.P., Goff L., Gupta S., Guy J., Harris W.P. (2020). Systemic Therapy for Advanced Hepatocellular Carcinoma: ASCO Guideline. J. Clin. Oncol..

[17] RAD51 Gene Family Structure and Function-PubMed. https://pubmed.ncbi.nlm.nih.gov/32663049/.

[18] Dynamics of DNA Double-Strand Breaks Revealed by Clustering of Damaged Chromosome Domains-PubMed. https://pubmed.ncbi.nlm.nih.gov/14704429/.

[19] DNA RECOMBINATION Base Triplet Stepping by the Rad51/RecA Family of Recombinases-PubMed. https://pubmed.ncbi.nlm.nih.gov/26315438/.

[20] BRG1 Promotes the Repair of DNA Double-Strand Breaks by Facilitating the Replacement of RPA with RAD51-PubMed. https://pubmed.ncbi.nlm.nih.gov/25395584/.

[21] Deng Y., Guo W., Xu N., Li F., Li J. (2020). CtBP1 Transactivates RAD51 and Confers Cisplatin Resistance to Breast Cancer Cells. Mol. Carcinog..

[22] Welsh J.W., Ellsworth R.K., Kumar R., Fjerstad K., Martinez J., Nagel R.B., Eschbacher J., Stea B. (2009). Rad51 Protein Expression and Survival in Patients with Glioblastoma Multiforme. Int. J. Radiat. Oncol. Biol. Phys..

[23] Zhang X., Ma N., Yao W., Li S., Ren Z. (2019). RAD51 Is a Potential Marker for Prognosis and Regulates Cell Proliferation in Pancreatic Cancer. Cancer Cell Int..

[24] Qiao G.-B., Wu Y.-L., Yang X.-N., Zhong W.-Z., Xie D., Guan X.-Y., Fischer D., Kolberg H.-C., Kruger S., Stuerzbecher H.-W. (2005). High-Level Expression of Rad51 Is an Independent Prognostic Marker of Survival in Non-Small-Cell Lung Cancer Patients. Br. J. Cancer.

[25] Mitra A., Jameson C., Barbachano Y., Sanchez L., Kote-Jarai Z., Peock S., Sodha N., Bancroft E., Fletcher A., Cooper C. (2009). Overexpression of RAD51 Occurs in Aggressive Prostatic Cancer. Histopathology.

[26] Richardson C., Stark J.M., Ommundsen M., Jasin M. (2004). Rad51 Overexpression Promotes Alternative Double-Strand Break Repair Pathways and Genome Instability. Oncogene.

[27] Klein H.L. (2008). The Consequences of Rad51 Overexpression for Normal and Tumor Cells. DNA Repair..

[28] TRIM36 Enhances Lung Adenocarcinoma Radiosensitivity and Inhibits Tumorigenesis through Promoting RAD51 Ubiquitination and Antagonizing Hsa-MiR-376a-5p-PubMed. https://pubmed.ncbi.nlm.nih.gov/36058131/.

[29] Liu Z., Huang J., Jiang Q., Li X., Tang X., Chen S., Jiang L., Fu G., Liu S. (2022). MiR-125a Attenuates the Malignant Biological Behaviors of Cervical Squamous Cell Carcinoma Cells through Rad51. Bioengineered.

[30] Targeting Rad51 as a Strategy for the Treatment of Melanoma Cells Resistant to MAPK Pathway Inhibition-PubMed. https://pubmed.ncbi.nlm.nih.gov/32719412/.

[31] Enhancing the Efficacy of Glycolytic Blockade in Cancer Cells via RAD51 Inhibition-PubMed. https://pubmed.ncbi.nlm.nih.gov/30183475/.

[32] Flygare J., Fält S., Ottervald J., Castro J., Dackland A.L., Hellgren D., Wennborg A. (2001). Effects of HsRad51 Overexpression on Cell Proliferation, Cell Cycle Progression, and Apoptosis. Exp. Cell Res..

[33] Chen J., Jin R., Zhao J., Liu J., Ying H., Yan H., Zhou S., Liang Y., Huang D., Liang X. (2015). Potential Molecular, Cellular and Microenvironmental Mechanism of Sorafenib Resistance in Hepatocellular Carcinoma. Cancer Lett..

[34] The DNA Damage Response: Ten Years after-PubMed. https://pubmed.ncbi.nlm.nih.gov/18082599/.

[35] Xu R., Yu S., Zhu D., Huang X., Xu Y., Lao Y., Tian Y., Zhang J., Tang Z., Zhang Z. (2019). HCINAP Regulates the DNA-Damage Response and Mediates the Resistance of Acute Myelocytic Leukemia Cells to Therapy. Nat. Commun..

[36] Targeting DNA Flap Endonuclease 1 to Impede Breast Cancer Progression. https://www.ncbi.nlm.nih.gov/pmc/articles/PMC5161424/.

[37] Wang C., Wang H., Lieftink C., du Chatinier A., Gao D., Jin G., Jin H., Beijersbergen R.L., Qin W., Bernards R. (2020). CDK12 Inhibition Mediates DNA Damage and Is Synergistic with Sorafenib Treatment in Hepatocellular Carcinoma. Gut.

[38] Samadaei M., Senfter D., Madlener S., Uranowska K., Hafner C., Trauner M., Rohr-Udilova N., Pinter M. (2022). Targeting DNA Repair to Enhance the Efficacy of Sorafenib in Hepatocellular Carcinoma. J. Cell Biochem..

[39] Chiu W.-C., Fang P.-T., Lee Y.-C., Wang Y.-Y., Su Y.-H., Hu S.C.-S., Chen Y.-K., Tsui Y.-T., Kao Y.-H., Huang M.-Y. (2020). DNA Repair Protein Rad51 Induces Tumor Growth and Metastasis in Esophageal Squamous Cell Carcinoma via a P38/Akt-Dependent Pathway. Ann. Surg. Oncol..

[40] FindFoci: A Focus Detection Algorithm with Automated Parameter Training That Closely Matches Human Assignments, Reduces Human Inconsistencies and Increases Speed of Analysis-PubMed. https://pubmed.ncbi.nlm.nih.gov/25478967/.

[41] Gyori B.M., Venkatachalam G., Thiagarajan P.S., Hsu D., Clement M.-V. (2014). OpenComet: An Automated Tool for Comet Assay Image Analysis. Redox. Biol..

[42] BRCA2 Is Required for Homology-Directed Repair of Chromosomal Breaks-PubMed. https://pubmed.ncbi.nlm.nih.gov/11239455/.

