# Supplementary material for: RAD51 Inhibition Shows Antitumor Activity in Hepatocellular Carcinoma
Source: Int J Mol Sci. 2023 Apr 26;24(9):7905. doi: 10.3390/ijms24097905 (PMC10178757; doi:10.3390/ijms24097905)
Supplement: Supplementary file 1 [file ijms-24-07905-s001.zip › ijms-2329779-supplementary.pdf]

RAD51 Inhibition Shows Antitumor Activity in Hepatocellular Carcinoma

Mingang Pan[1, †], Yu Sha[1,2, †], Jianguo Qiu[3], Yunmeng Chen[1], Lele Liu[1], Muyu Luo[1], Ailong Huang[1*], Jie Xia[1]**

1 Key Laboratory of Molecular Biology for Infectious Diseases (Ministry of Education), Chongqing Medical University, 400016, Chongqing, China

2 Henan University of Chinese Medicine, Zhengzhou, 450000, Henan, China.

3 Department of hepatobiliary surgery, The First Affiliated Hospital, Chongqing Medical University, 400016, Chongqing, China

* Corresponding author. E-mail: ahuang@cqmu.edu.cn; Tel: +86 23 68486780; Fax: +86 23 68486780;

** Corresponding author. E-mail: xiajie@cqmu.edu.cn; Tel: +86 23 68486780; Fax: +86 23 68486780;

† These authors contribute equally to this work.

Supplementary Figures

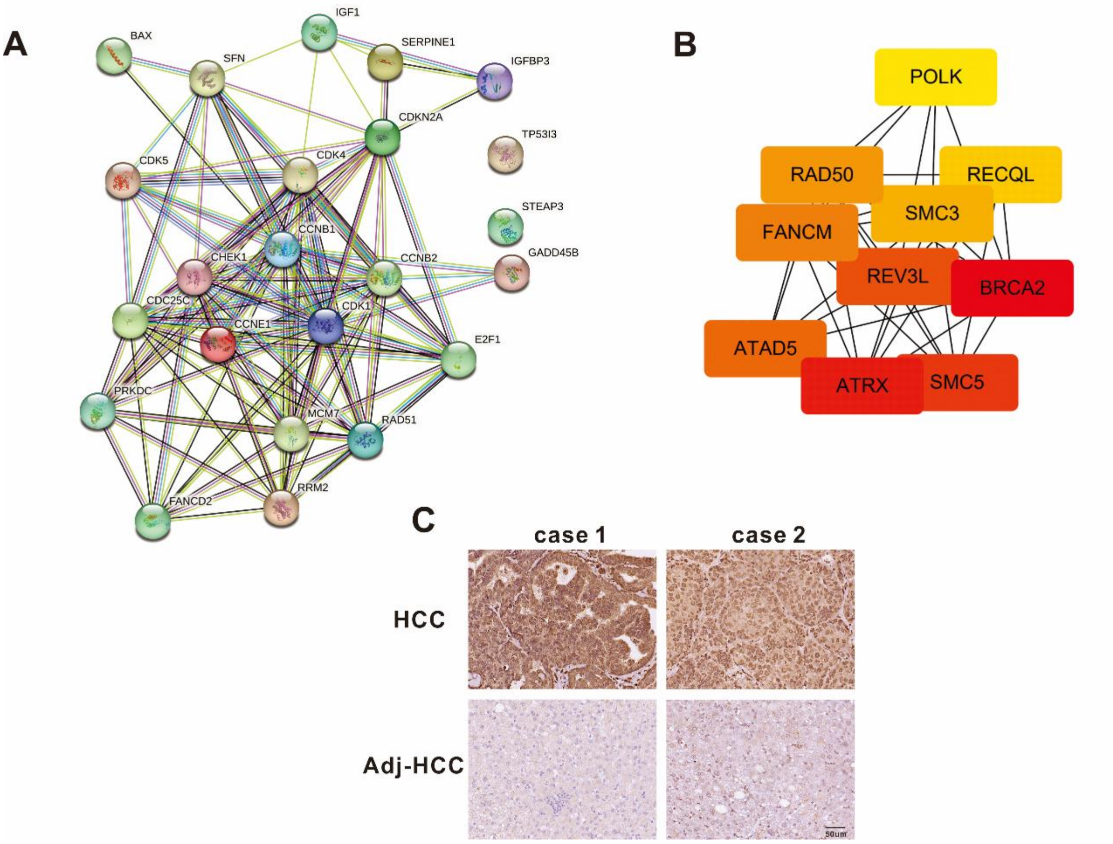

Figure S1. Identified RAD51 as a potential DDR target for HCC, and RAD51 expression was upregulated in HCC. (A) PPI network of the intersection genes of LIHC differential genes and the DDR genes was generated by STRING database. (B) The top 10 hub genes were calculated using the cytoHubba plugin of Cytoscape software. (C) RAD51 protein expression was detected using immunohistochemical staining, and representative samples were presented.

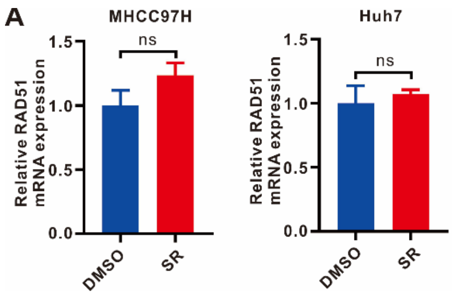

Figure S2. Sorafenib didn't decreased RAD51 mRNA expression in MHCC97H and Huh7 cells. (A)MHCC97H and Huh7 cells were treated with sorafenib at 5 uM and 3 uM respectively. After 24h, RAD51 mRNA was detected using RT-qPCR. Experiment was performed thrice independently with three replicates for each experiment. ns, no significant.

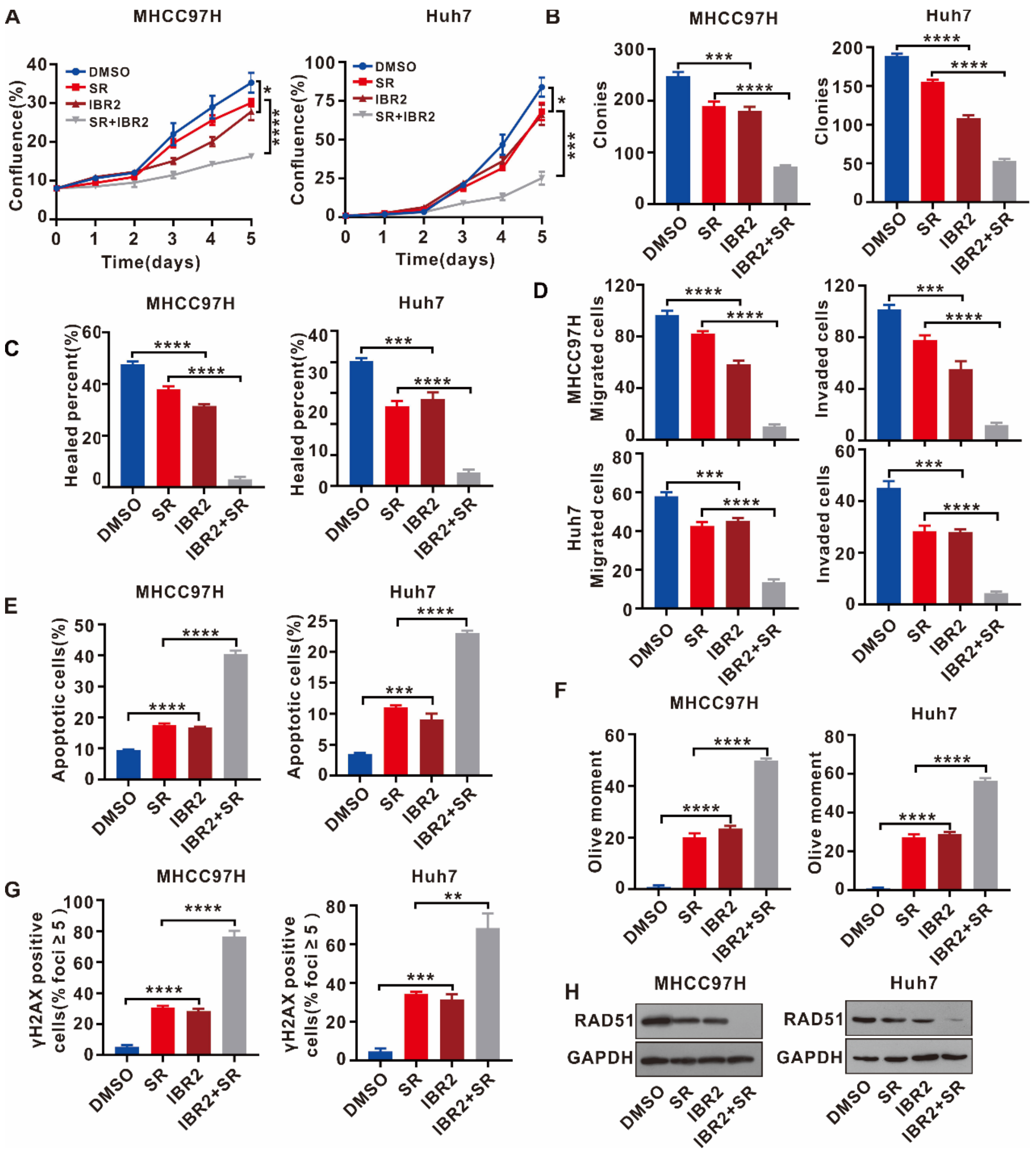

Figure S3. IBR2 shows a synergistic antitumor effect with sorafenib. (A) The effect of IBR2 combined with sorafenib on cell proliferation of HCC cells was determined using the IncuCyte cell proliferation assay. (B) Investigating the combined effect of IBR2 and sorafenib on HCC cell's colony formation ability using colony formation assays. (C) Wound healing assays were used to evaluate the effect of IBR2 combined with sorafenib on cell migration. (D) The effect of IBR2 combined with sorafenib on cell migration and invasion was detected using transwell assays. (E) Cell apoptosis was analyzed to evaluate the effect of IBR2 combined with sorafenib on HCC cells. (F) DNA damage of HCC cells treated with IBR2 or sorafenib was examined using the comet assay. (G) The level of γH2AX was examined in MHCC97H and Huh7 cells treated with sorafenib or RAD51 siRNA using immunofluorescence. (H) The combined effect of IBR2 with sorafenib on RAD51 protein levels was detected using WB assays. MHCC97H cell were treated with sorafenib at 5 uM or/and IBR2 at 20 uM, Huh7 cell were treated with sorafenib at 3 uM or/and IBR2 at 20 uM in these assays. Each experiment was performed thrice

independently with three replicates for each experiment. All *P < 0.05, **P < 0.01, ***P < 0.001, ****P < 0.0001

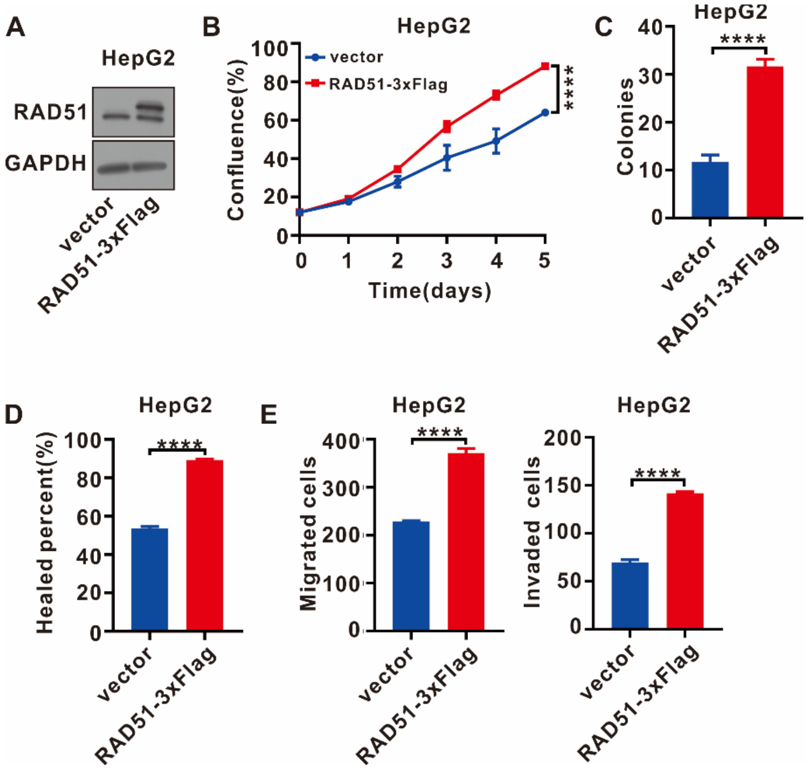

Figure S4. RAD51 overexpression increased cell proliferation, migration, and invasion. (A) Overexpression of RAD51 in HepG2 cells and the overexpression effect was assessed using WB assay. (B) The effect of RAD51 overexpression on cell proliferation was evaluated by IncuCyte cell proliferation assays. (C) HepG2 Cells with RAD51 overexpression were grown for two weeks, and the colonies were captured and counted. (D) Wound healing assays were used to investigate the effect of RAD51 overexpression on cell migration. (E) Transwell assays were conducted to assess the effect of RAD51 overexpression on cell migration and invasion. Each experiment was performed thrice independently with three replicates for each experiment. All *P < 0.05, **P < 0.01, ***P < 0.001, ****P < 0.0001
